# Spontaneous phase separation and pattern formation in a lyotropic nematic mixture

**DOI:** 10.1073/pnas.2604649123

**Published:** 2026-07-09

**Authors:** Ana Bensabat, Órlaith Skelton, Jochen Arlt, Marko Bjelogrlić, Davide Marenduzzo, Giuseppe Negro, Tyler N. Shendruk, Tiffany A. Wood

**Affiliations:** ^a^ School of Physics and Astronomy, University of Edinburgh, Edinburgh EH9 3FD, United Kingdom

**Keywords:** phase separation, liquid crystals, chromonics, lyotropic nematics, self-assembled smectics

## Abstract

Lyotropic liquid crystals can display rich phase behavior and self-organization, yet the physical principles underlying their self-assembly into large scale patterns remains understudied. Here, we combine theory, simulations, and experiments on Sunset Yellow-water chromonic mixtures to show that such materials spontaneously phase separate, even without assuming any underlying microscopic attraction between the molecular species. In our minimal model, demixing depends solely on the Onsager-like coupling between local nematogen density and orientational order. If such a coupling is sufficiently strong, nematic defects trigger the nucleation of isotropic droplets, which then coalesce due to elastic or interfacial tensions. We further show that strong anchoring of the director field at the interface arrests this coarsening process, resulting in a stable microphase-separated lamellar pattern. This self-assembled lamellar phase has striking and unusual features, including spontaneous undulations, heterogeneous layer spacing, long-lived glassy defect patterns and lamellar onions. Our results identify orientational-density coupling and elastocapillarity as fundamental mechanisms to guide self-assembly in lyotropic and chromonic liquid crystals.

## Significance

Liquid crystals are widely used in technology, yet their behavior in mixtures with simple liquids remains ill-understood, because molecular orientation, elasticity, and interfaces interact in complex ways. Chromonic materials such as Sunset Yellow display robust phase separation, even though there is no strong attraction between their molecules. Here we show that this demixing can arise solely from the coupling between local concentration and orientational order, which nucleates isotropic droplets at nematic defects that later on coalesce due to elastic or capillary forces. Our results also reveal the emergence of unusual lamellar phases with undulations and long-lived defect patterns. These mechanisms are generic to many soft materials, offering a route to engineer self-organized biocompatible and responsive composite materials.

Lyotropic liquid crystals (or simply lyotropics) are composite materials with surprisingly rich phase behavior and potential for self-assembly (1). Besides being of fundamental scientific interest, they are also appealing technologically, because they can be used as biosensors (2), or as metamaterials with unusual optical, flow or mechanical properties (3).

Chromonic liquid crystals provide a fascinating example of lyotropics. They consist of molecules that reversibly stack into aggregates stabilized by aromatic interactions (4, 5). Their biocompatibility together with the ability to form multiple self-assembled phases make them promising candidate vessels for targeted drug delivery and biological sensing (6). Sunset Yellow (SSY) is one of the most widely studied chromonics because it readily forms lyotropic nematics and is amenable to experimental investigation (7). Yet, despite this attention, the physical origin of the well-documented broad nematic–isotropic coexistence region in SSY–water mixtures (4, 7–10) remains poorly understood. In particular, there is little evidence that SSY molecules experience strong mutual attraction, raising the question of what controls the observed phase separation.

From a fundamental physics viewpoint, mixtures of liquid crystalline and isotropic fluids exhibit strikingly nontrivial emergent behavior (11–13), due to the interplay between elasticity, anchoring, and interfacial stresses. This behavior can be captured by several key dimensionless numbers, such as the elastocapillary number determining the balance between elastic and interfacial stress (12), and the ratio between anchoring strength and surface tension (14). The balance between anchoring strength and elasticity is also well-known to determine the defect structure around inclusions (15–20), or the self-assembly of colloids and defects at air–water interfaces (21). The diversity of patterns and richness in behavior observed across lyotropics arises because these dimensionless ratios can take on a wide range of values, corresponding to different balances between the associated forces, and hence to different physical regimes.

In this work, we combine computer simulations and theory to study phase separation and pattern formation in a lyotropic system; we also compare the predicted phase behavior with what we observe experimentally in mixtures of SSY and water at different compositions and temperatures. The theoretical free energy functional which we use favors the mixed phase in the absence of liquid crystalline order—in other words, we do not assume any attractive interactions between the molecular species which would lead to demixing. First, such a minimal model for a lyotropic mixture is useful because the results are likely to apply generally, to a large class of materials. Second, this premise is a reasonable simple starting point for SSY, where the constituent molecules have charged sulfonate groups at their periphery, leading to electrostatic repulsion between self-assembled SSY stacks, so that any effective like-charge attraction between stacks would likely need to result from more complicated fluctuation-induced many-body interactions (see the discussion in ref. 4). An important result we obtain with our minimal model is that the Onsager-like coupling between local nematogen density and orientational order favors the formation of isotropic droplets at nematic defects, which nucleates spontaneous phase separation. This provides a robust mechanism to rationalize the generic presence of a sizable phase separation region in the phase diagram of SSY, without the need to assume any significant affinity between SSY stacks.

Our theory also predicts that coarsening is arrested and the system forms a lamellar system when the anchoring of the nematic director at interfaces—either tangential or normal—is sufficiently strong. This phase is also observed experimentally in our chromonic SSY mixture. Unlike smectics, this self-assembled structure is characterized by pronounced undulations, which lead to the formation of long-lived, and possibly glassy, defect patterns, including edge dislocations and lamellar onions. While the lamellar size is self-selected and well-defined, the spacing between layers is heterogeneous and uneven, suggesting that the layer-compression modulus of our self-assembled phase-separated stripes is unusually small. This property may be the reason why onions form in our system, in contrast with smectics where these patterns are rare, especially in the absence of a shear flow (22). Additionally, the fact that this lamellar phase contains essentially frozen defect patterns which depend on initial condition and sample history suggest that lyotropic mixture, such as SSY, may provide an understudied example of multistable self-assembled and tunable glass with biocompatible properties.

## Spontaneous Phase Separation in Lyotropics

### Experimental and Simulation Phase Diagrams for Lyotropics Show Broad Coexistence Regions.

**Fig. 1.**
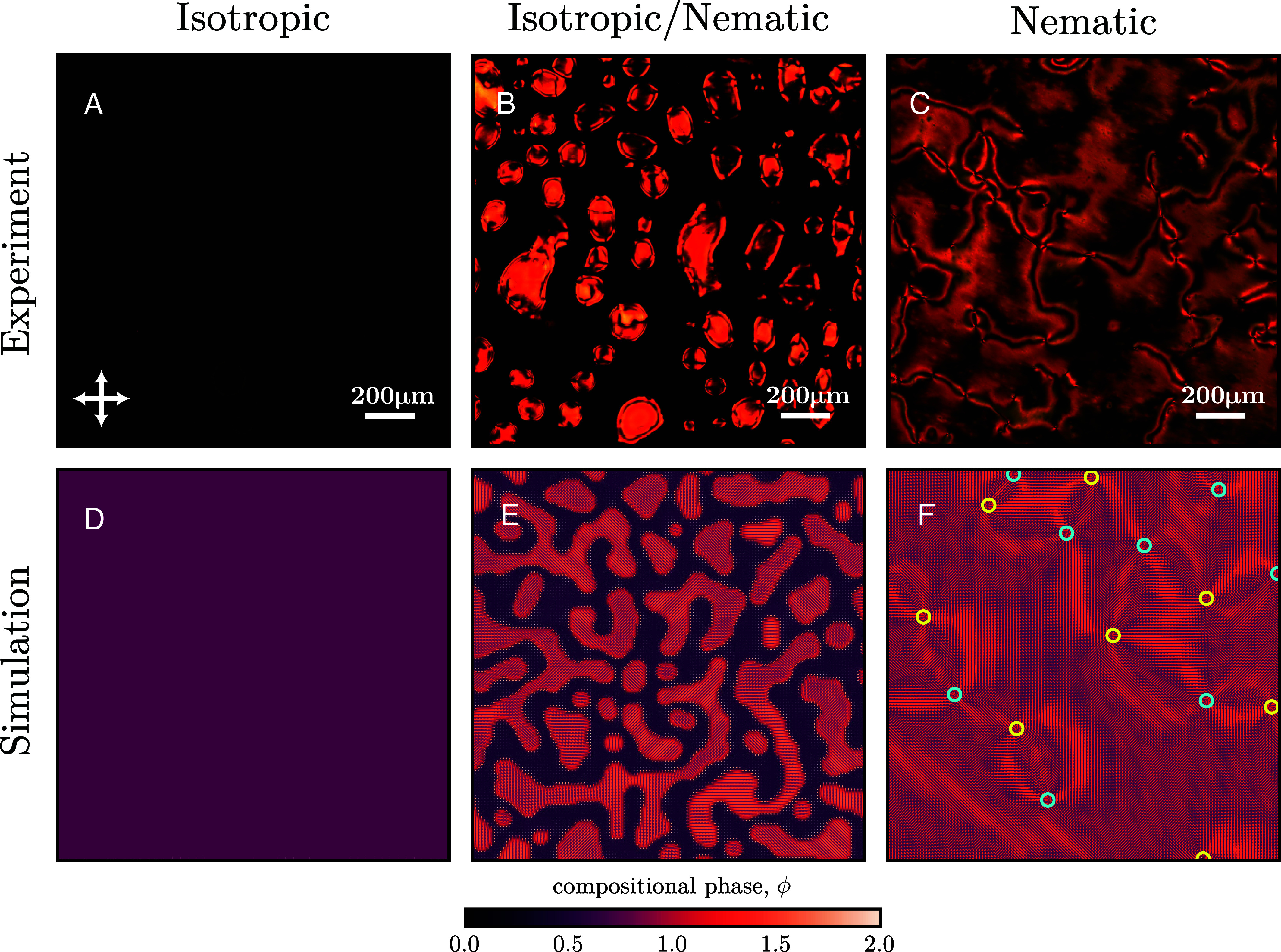
Simulations replicate the isotropic–nematic (I–N) transition and phase separation. (*A*–*C*) Experimental images showing the isotropic (*A*), coexistence (*B*), and nematic (*C*) phases as the temperature decreases ($\gamma_{0}$ increases) and the nematic fluid concentration $\phi_{0}$ increases. (*D*–*F*) Corresponding simulation snapshots illustrating the same sequence of phase behavior for a 128 × 128 system. The sets of parameters $\left(\phi_{0},\gamma_{0}\right)=\left(0.7,1.7\right)$, (0.9,1.9) and (1.4,2.4) were used for the isotropic (*D*), coexistence (*E*), and nematic (*F*) phases, respectively. The color map represents the local compositional phase, ϕ, while the overlaid lines indicate the nematic director field, with line length proportional to the local degree of orientational order. The cyan and yellow rings represent topological defects of charge +1/2 and −1/2, respectively. (Scale bars: 200 μm.) Vertical and horizontal arrows in panel (*A*) show the polarizer and analyzer orientations, respectively.

We start by reporting results on the experimental phase behavior of SSY-water mixture (see *Materials and Methods* for details of materials preparation, and free energy functional used in the simulations). Our experiments show that, by changing composition and temperature, three regimes can be readily observed: an isotropic phase (Fig. 1*A*), a nematic phase (Fig. 1*C*), and an intermediate phase-separated regime, where isotropic and nematic domains coexist (Fig. 1*B*). This coexistence region is striking because SSY monomers should in principle exhibit electrostatic repulsion between them due to the sulfonate groups (10) suggesting that a different mechanism other than an underlying thermodynamic attraction between SSY stacks may drive demixing.

To explain the phase separation observed experimentally we introduce a minimal Landau–de Gennes model for lyotropic liquid crystals in which the liquid crystalline component of the free energy density depends on the nematic tensor order parameter Q (see *Materials and Methods* for its definition) as

[1] $$\begin{array}{c} \begin{array}{cc} f_{\mathrm{LC}}= & \frac{A_{0}}{2}\left(1-\frac{\gamma }{3}\right)Q_{\alpha \beta }^{2}-\frac{A_{0}\gamma }{3}Q_{\alpha \beta }Q_{\beta \gamma }Q_{\gamma \alpha }\\ & +\frac{A_{0}\gamma }{4}{\left(Q_{\alpha \beta }^{2}\right)}^{2}+\frac{K}{2}{\left(\partial_{\alpha }Q_{\beta \gamma }\right)}^{2}. \end{array} \end{array}$$

In Eq. **1**, the polynomial terms account for the bulk free energy density, where $A_{0}$ is a positive constant, while the final term accounts for the elastic energy associated with the director field, governed by the constant K. The free energy density of the binary mixture is given by

[2] $$\begin{array}{c} f_{\phi }=\frac{a}{2}\phi^{2}+\frac{\kappa }{2}{\left(\partial_{\alpha }\phi \right)}^{2}+WQ_{\alpha \beta }\partial_{\alpha }\phi \partial_{\beta }\phi , \end{array}$$

where a is a positive constant. We highlight that, in contrast with previous works (23, 24), the first term is a single well describing the binary mixture in the mixed phase, which on its own cannot result in phase separation. The second term defines the interfacial energy. The third term couples the orientational order parameter to compositional gradients, enforcing preferential anchoring at interfaces (further details in *Materials and Methods*).

Remarkably, even our minimal model for lyotropic liquid crystals, which contains no anchoring term (W=0), no explicit interfacial tension (κ=0), and no attractive interactions (explicit demixing terms are absent from the bulk free energy), reproduces all three regimes observed experimentally (Fig. 1*D*–*F*). In the phase-separated region, the system self-organizes into a number of nematic rafts which are not circular, suggesting that elasticity leads to the emergence of an effective weak, or anisotropic, surface tension. In the simulations, the rafts exhibit sharper features due to the omission of explicit interfacial tension. Reintroducing surface tension results in smoother, rounded morphologies that more closely match experimental observations (Fig. 3 *A* and *B*). These nematic rafts tend to be internally ordered and defect-free in simulations (Fig. 1*E*); experimentally, some defects remain as apparent from the Schlieren patterns in Fig. 1*B*, possibly reflecting the presence of some nonnegligible anchoring in the experiments. Defects are instead transiently observed in simulations in the nematic regime (Fig. 1*F*), which is qualitatively consistent with experimental observations (Fig. 1*C*).

**Fig. 2.**
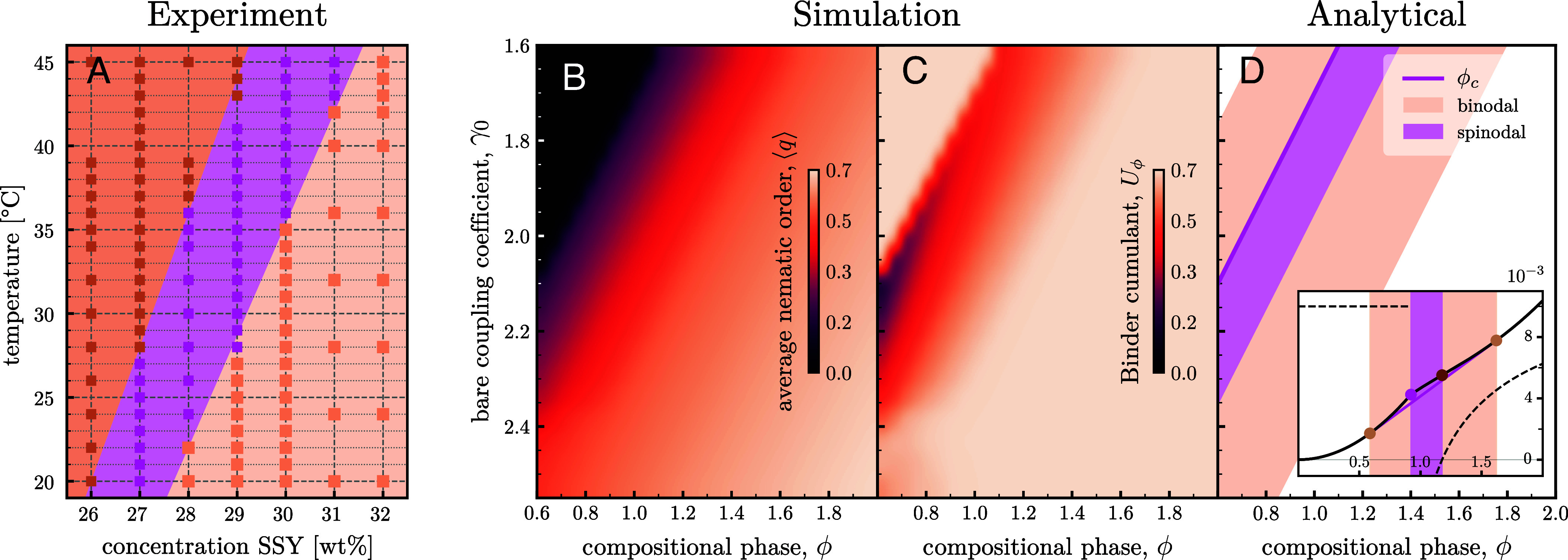
Experimental, simulated, and analytical phase diagrams reveal the isotropic–nematic transition and coexistence regions. (*A*) Experimental phase diagram of SSY solutions showing isotropic (brown), coexistence (violet), and nematic (beige) regions as functions of concentration and temperature. (*B* and *C*) Simulated phase behavior represented by the average nematic order ⟨q⟩ and Binder cumulant, $U_{\phi }$, as functions of bare coupling coefficient $\gamma_{0}$ and composition ϕ. (*D*) Analytical phase diagram derived from the common tangent construction on the free-energy density $f_{\text{hom}}$, showing the binodal and spinodal limits. The pink line denotes $\phi_{c}$, the nematic fluid concentration marking the transition from a homogeneous to a phase-separated configuration. *Inset*: Schematic of the common tangent construction, plotting the homogeneous free energy density $f_{\text{hom}}$ (solid line) and its second derivative $\partial_{\phi }^{2}f_{\text{hom}}$ (dashed lines) as a function of ϕ. The tangent points (beige circles, $\phi_{\pm }$) define binodal limits, while regions of negative curvature define spinodal boundaries (bounded by the pink circle, denoting the discontinuity in $\partial_{\phi }^{2}f_{\text{hom}}$, and the brown circle), indicating that phase-separated states are energetically favored over homogeneous ones.

More quantitatively, we systematically map the phase diagram of our chromonic SSY mixture in our experiments for different values of composition (26 to 32 Wt%) and temperature (20 to 45 °C). The phase diagram features linear phase boundaries and a broad coexistence region (Fig. 2*A*). The phase diagram is in good agreement with those found in the literature for SSY mixtures (7–9). Within the coexistence region, the ratio of nematic to isotropic domains changes with temperature, an example of which is shown in *SI Appendix*, Fig. S2.

Our minimal model for lyotropics leads to a phase diagram which is similar in shape (Fig. 2 *B* and *C*), where phases are identified by measuring the average nematic order and the Binder cumulants computed from the probability distribution functions for local composition ϕ (*SI Appendix*). Unlike conventional LC mixtures, which show curved binodals typical of attraction-driven demixing (11, 23–25), both the experimental SSY–water mixture and the minimal model exhibit nearly linear phase boundaries, suggesting that these mixtures are qualitatively different from those considered theoretically in the past, and that the functional form employed for the free energy potential in our model is the appropriate one for our experimental system.

### Coupling-Induced Phase Separation.

To explain the phase diagram observed in both experiments and simulations, we analyze the equilibrium thermodynamics of the binary mixture. Within our model, the bulk free energy (Eq. **1**) is coupled to the composition ϕ through an effective inverse reduced temperature $\gamma \left(\phi \right)=\gamma_{0}+\Delta \;\phi$, where Δ is the coupling parameter. The uncoupled system (Δ=0) mixes freely implying that the phase separation observed here is driven entirely by the coupling between the composition, ϕ, and the nematic order, whose magnitude is q. Our theory and simulations for the coupled system use Δ=1; different values of Δ>0 give qualitatively similar results.

We determine the phase boundaries using the common tangent construction (26), applied to the free energy of a homogeneous configuration of unvarying composition and uniformly aligned nematic phase. In this state, the elastic term vanishes (Eq. **1**), and the homogeneous free energy density depends only on the nematic order q and the composition ϕ as

[3] $$\begin{array}{c} \begin{array}{cc} f_{\text{hom}}\left(q,\phi \right)= & \frac{A_{0}}{3}\left(1-\frac{\gamma }{3}\right)q^{2}-\frac{2A_{0}\gamma }{27}q^{3}\\ & +\frac{A_{0}\gamma }{9}q^{4}+\frac{a}{2}\phi^{2}. \end{array} \end{array}$$

In this homogeneous configuration, the local nematic density is constant in space, ϕ(r)=⟨ϕ⟩ for all locations r. The total free energy is thus minimized for the nematic order

[4] $$\begin{array}{c} q_{\text{min}}\left(\phi \right)=\left\{\begin{array}{cc} 0, & \text{if}\;\gamma \leq \gamma_{c}\;,\\ \frac{1}{4}\left(1+\sqrt{9-24/\gamma (\phi )}\right), & \text{if}\;\gamma \geq \gamma_{c}. \end{array}\right. \end{array}$$

The homogeneous configuration is stable against phase decomposition only if the corresponding free energy, $f_{\text{hom}}\left(q_{\text{min}},\phi \right)$, is convex. However, when coupling between compositional and orientational order is considered, the free energy can become concave, defining a spinodal region where the second derivative of the free energy is negative and the mixture is unstable (Fig. 2*D*, *Inset*). The stable coexisting phases (binodals) are identified via the common tangent construction using an iterative method to find the unique tangent line at two distinct concentrations, $\phi_{-}$ and $\phi_{+}$. This line represents the average free energy density of the phase-separated mixture, which is lower than that of the homogeneous state for any given average composition ⟨ϕ⟩ lying between the binodals. The spinodal region determined semi-analytically is in agreement with the phase separation regions mapped in our experiments and simulations (Fig. 2); it also compares well with that found in other experimental works (7–9).

### Elastic and Interfacial Stresses Give Different Demixing Dynamics.

**Fig. 3.**
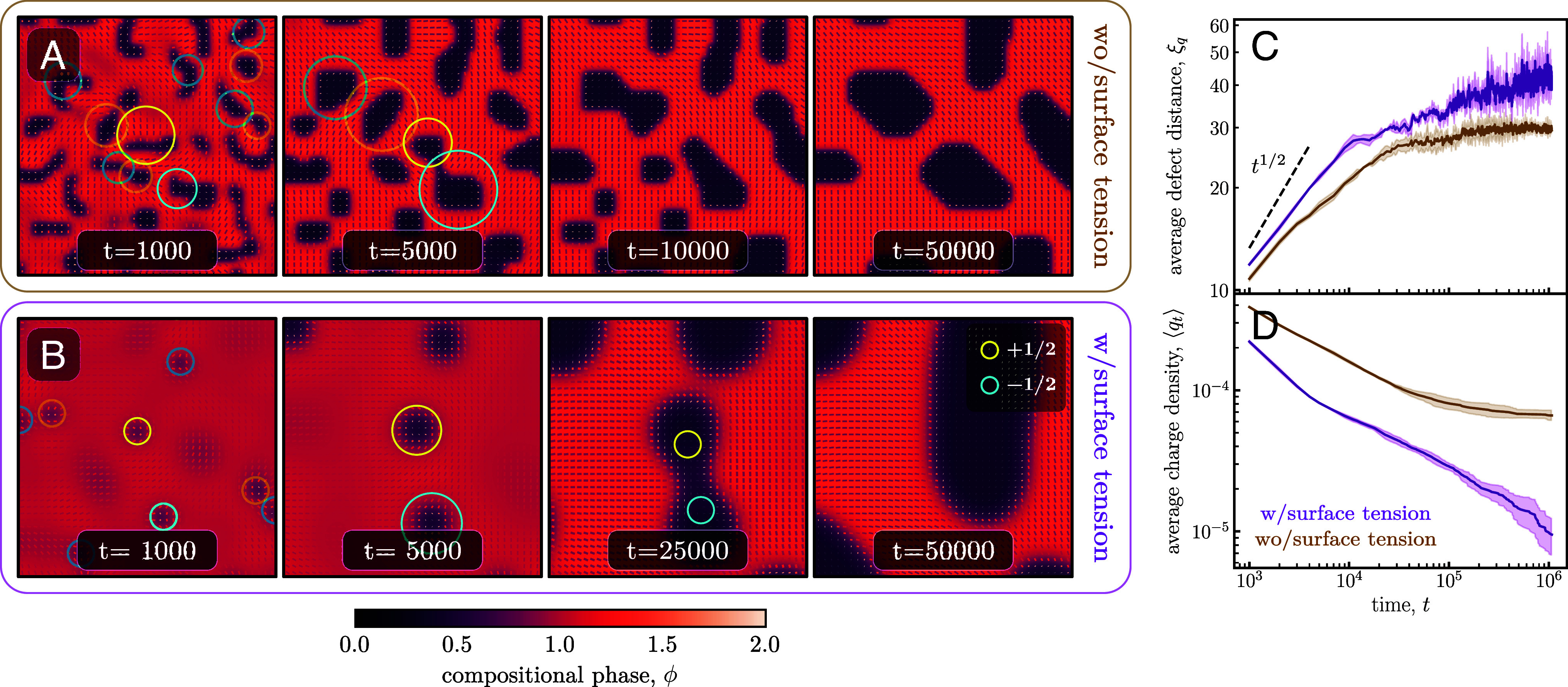
Defect-driven phase separation and droplet coarsening dynamics with and without surface tension. Starting from a random, noisy initial configuration, the nematic field evolves toward macroscopic phase separation through the annihilation of +1/2 (yellow) and −1/2 (blue) topological defects, leading to the formation of isotropic voids that grow and coarsen over time. Panel (*A*) shows a simulation without surface tension (κ=0.00) and panel (*B*) includes surface tension (κ=0.01). The column on the *Right* shows the average defect distance over time, $\xi_{q}$ (*C*) and the average charge density, $q_{t}$ (*D*). Both quantities are averaged over three runs with different randomized initial configurations in which the mean compositional phase is fixed at $\phi_{0}=1$ and the bare coupling coefficient at $\gamma_{0}=2.1$. Both A and B show magnified views (40×40) of the global simulation domain (128×128). The full animations are available in Movies S1 and S2. A qualitatively similar coarsening is observed experimentally when a mixture of SSY at 28 wt% undergoes a temperature ramp (*SI Appendix*, Fig. S2 and Movie S3).

While the previous theory indicates whether or not the system is unstable to demixing, it does not reveal how phase separation proceeds dynamically, neither does it predict whether the end result is macroscopic phase separation or microphase separation. To address these two questions, we have used simulations to follow the demixing dynamics (Fig. 3).

We first consider the case of a lyotropic mixture with negligible interfacial tension (σ=0) between the two fluids (Fig. 3*A*; *Materials and Methods*). Starting from a noisy nematic phase, nucleation of defects with topological charge ±1/2 occurs. Instead of annihilating, as would occur in a single-phase nematic fluid, these defects first nucleate a local change in composition resulting in the formation of small isotropic voids where the defects were (Fig. 3*A*). As the nematic director near the droplet interface retains memory of the defect patterns, elastic stresses drive the coalescence of neighboring droplets which have opposite value of local topological charge. As in a single-phase nematic (27), the number of defects, or more precisely the integral of the modulus of the topological charge density (*Materials and Methods*) decreases over time, because such defects introduce an elastic stress in the material which is proportional to the elastic constant K. The decrease in number of defects causes the average distance between defects to increase with the square root of time, $\xi_{q}∼t^{1/2}$ (Fig. 3*C*), following the scaling law governing defect coarsening in single-phase nematics (27). Concomitantly, we observe that the average absolute charge density $\langle q_{t}\rangle$ decreases. In the lyotropic mixture, this topological charge annihilation process drives droplet coalescence, and because of the decrease in the overall topological charge with time, this coalescence-based coarsening slows down dramatically and appears to arrest at late times, with the average absolute charge density plateauing to small nonzero values at long times (Fig. 3*D*). This kinetic arrest explains the microphase separation observed in our simulations for negligible surface tension.

For sufficiently strong interfacial tension σ, the growth of isotropic domains proceeds in a qualitatively different way (Fig. 3*B*). The driving force for coalescence is now provided by both elasticity and the interfacial tension; as a result, coarsening proceeds indefinitely (Fig. 3 *C* and *D*), leading to macroscopic phase separation in steady state.

We suggest that the dynamical crossover between elastically-driven-then-arrested coarsening and interfacially-driven macrophase separation is controlled by the elastocapillary number Ec controlling the balance between interfacial and elastic stresses (12), which in our minimal model can be written simply as Ec=κ/K, where κ is related to the interfacial energy and K is the nematic elastic constant (*Materials and Methods*). When Ec is small, coarsening is driven by elasticity and interaction between local residual topological charges, whereas coarsening is driven by surface tension and resembles standard binary fluid phase separation when Ec is large (28). The dimensionless elastocapillary number is Ec=0 in Fig. 3*A* and Ec=1 in Fig. 3*B*. This trend holds across different values of K, as illustrated in *SI Appendix*, Figs. S4 and S5; the interplay between interfacial and elastic stresses therefore spans a range of behaviors that can emulate different chromonic mixtures. We note that a sufficiently large nematic elasticity K provides an effective contribution to the surface tension that aids coarsening. Therefore, for large K, the definition of the elastocapillary number should be given in terms of an effective surface tension $\tilde{\kappa }$ which includes elastic effects.

## Pattern Formation in Lyotropic mixtures

### Anchoring Induces the Self-Assembly of Microphase-Separated Stripes.

**Fig. 4.**
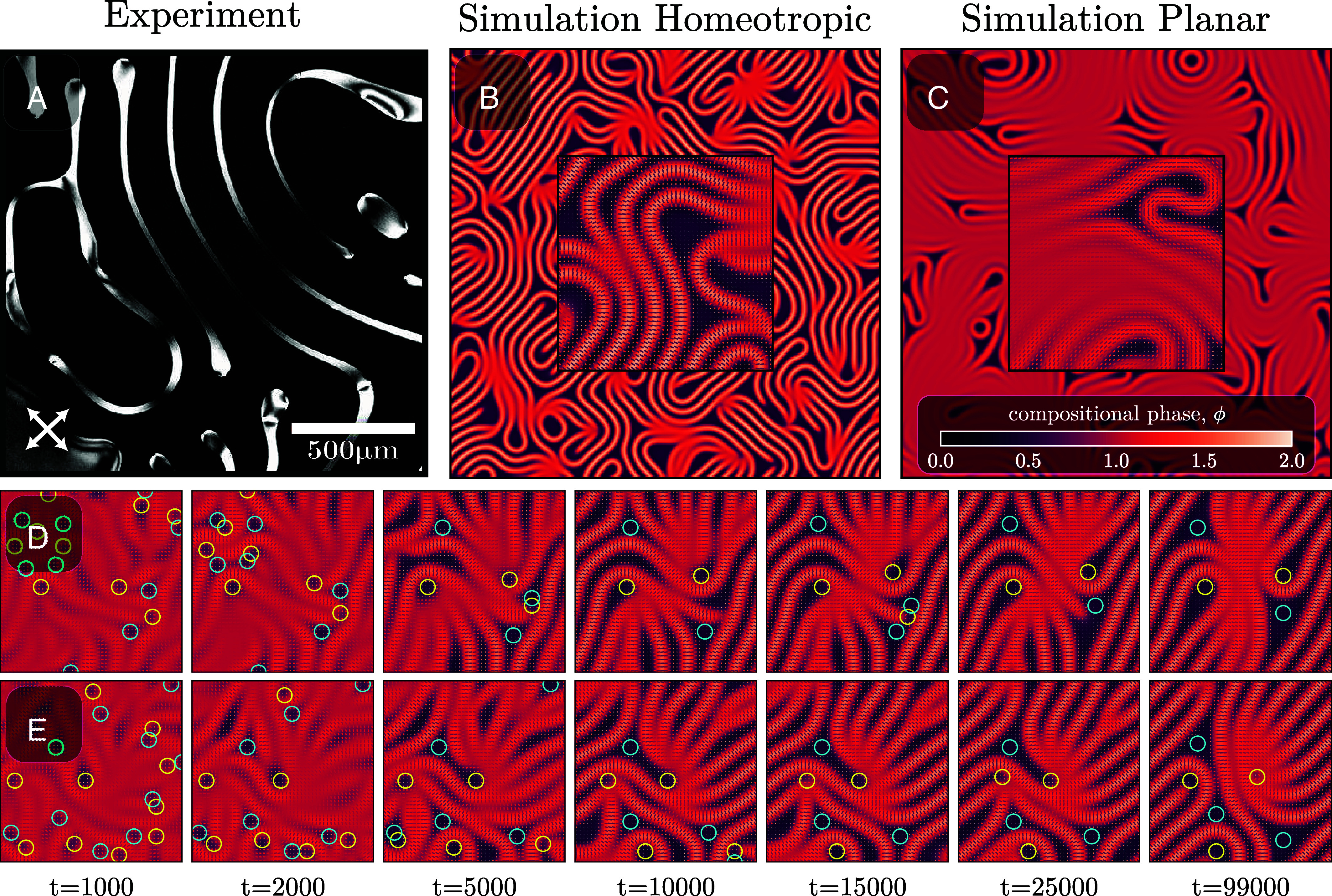
Anchoring-induced microphase separation and lamellar ordering in the isotropic–nematic mixture. (*A*) Experimental snapshot showing phase separation into alternating nematic and isotropic layers (see Movie S4 for the complete dynamics). (*B* and *C*) Corresponding simulations exhibiting similar lamellar structures, albeit more densely packed. Simulation parameters are $\gamma_{0}=2.0$, $\phi_{0}=1.0$, and anchoring strength W=−0.03 (*B*), corresponding to normal anchoring, and W=0.06 (*C*), corresponding to planar anchoring. (*D* and *E*) Time evolution of two simulations with different initial conditions (same parameters as in *B*) showing defect dynamics: +1/2 (yellow) and −1/2 (blue) defects appear, move, and annihilate to relax the lamellar structure (Movies S5 and S6). In some cases, defects reappear transiently (*D*), while in others, a persistent +1/2 defect acts as a source of nematic layers (*E*). In both cases, complete annihilation is inhibited, leading to arrested, anchoring-stabilized microphase separation. Arrows in panel *A* show the polarizer and analyzer orientations.

The results discussed up to now can be understood within a model which does not include any interfacial anchoring of the director field. Introducing anchoring—either normal or tangential—leads to additional physics and pattern formation (Fig. 4 and Movies S5 and S6).

In particular, as the anchoring strength W increases past a critical threshold, a completely different and previously unexplored phase self-assembles starting from a uniform disordered phase with noise. This is a self-assembled lamellar phase that is observed experimentally (Fig. 4*A* and Movie S4), close to the transition from coexistence to the isotropic phase. This lamellar phase is also observed in simulations (Fig. 4*B*).

In this lamellar phase, the domains consist of elongated layers of microphase-separated nematic domains. Unlike the lamellar phases seen in lyotropics and copolymers (29), these stripes are stabilized by anchoring along the interfaces of the layer-like microphase-separated domains.

To understand more quantitatively the mechanism driving the self-assembly of the lamellae, we examine the thermodynamic stability of a single lamellar nematic domain with a well-defined interface. We consider a region where the director field n(r) is spatially uniform and coupled to the domain interface. The director n can either be parallel to the layer normal direction ∇ϕ (homeotropic anchoring) or perpendicular to ∇ϕ (planar anchoring). Assuming that the local nematic order is linearly coupled to the local concentration via q(r)≈αϕ(r), the free energy density can be recast to isolate the gradient contributions

[5] $$\begin{array}{c} \begin{array}{c} f_{\text{lam}}\approx f_{\text{bulk}}\left(\phi ,q\right)+\kappa_{\text{eff}}\left(\phi ,q\right){\left(\nabla \phi \right)}^{2}, \end{array} \end{array}$$

where an effective surface tension coefficient

**Fig. 5.**
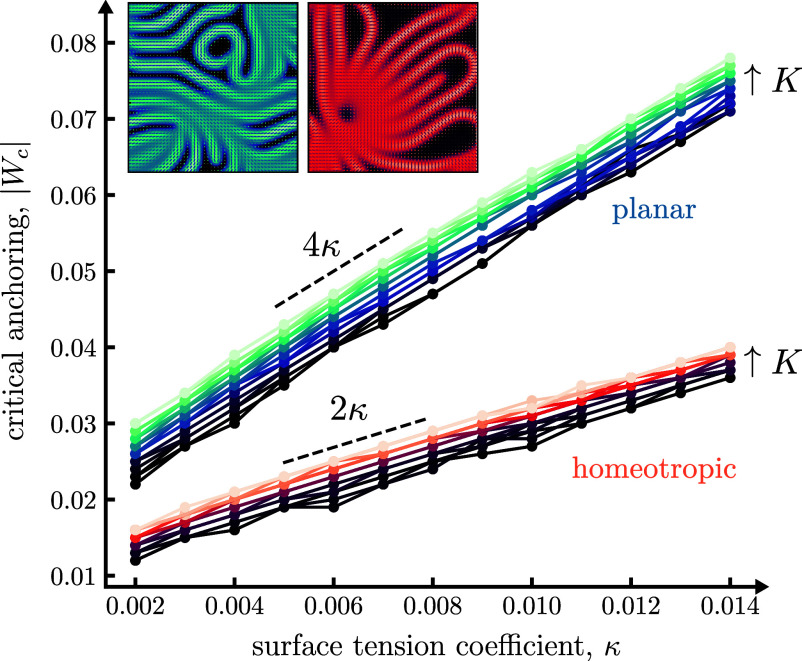
Critical anchoring for planar and homeotropic cases. The critical anchoring follows the theoretical predictions (dashed lines) for both planar (blue) and homeotropic (orange) anchoring as a function of the surface tension coefficient, κ. Line brightness increases as a function of the elastic nematic constant, K, varied from 0.10 to 0.30. *Insets Top Left* show typical system configurations at critical anchoring strength $|W_{c}|$.

[6] $$\begin{array}{c} \kappa_{\text{eff}}=\frac{\kappa }{2}+\frac{K\alpha^{2}}{3}+Wq\left(\frac{{\left|n\cdot \nabla \phi \right|}^{2}}{{\left|\nabla \phi \right|}^{2}}-\frac{1}{3}\right) \end{array}$$

appears. The last term in $\kappa_{\text{eff}}$ captures an asymmetry between homeotropic and planar critical anchoring, which is observed in the simulations (Fig. 5).

Macrophase separation is driven by a positive surface tension ($\kappa_{\text{eff}}>0$), which drives the system to minimize interfacial area. However, for both types of anchoring, the last term in $\kappa_{\text{eff}}$ (Eq. **6**) is negative and so a transition occurs when this anchoring contribution renders $\kappa_{\text{eff}}$ negative. In this regime, the creation of interfaces is in fact favored: The mechanism leading to microphase separation is therefore effectively the same as that of Brazovskii theory (30–32), although in our case it is the anchoring which renders the surface tension effectively negative.

In the supersmectic phase, maximizing interface contour length while minimizing surface tension is achieved via long stacked lamellae. The presence of the lamellae interrupts pathways to the global free energy minimum, which arrests the coarsening process and stabilizes smaller length scales characteristic of microphase separation. The critical threshold for this transition ($\kappa_{\text{eff}}=0$) is defined by

[7] $$\begin{array}{c} \left|W_{c}^{⊥}\right|=\frac{3}{2q}\left(\frac{\kappa }{2}+\frac{K\alpha^{2}}{3}\right),\;W_{c}^{\|}=2\left|W_{c}^{⊥}\right| \end{array}$$

for homeotropic and planar anchoring, respectively. Evaluating these expressions at the center of the interface between the nematic *lamellae* and the surrounding isotropic fluid makes a quantitative prediction for the critical values. We approximate the local composition as the midpoint between the nematic phase (ϕ≈1.5 in the simulations) and the isotropic fluid (ϕ≈0.5 in the simulations), yielding ϕ≈1. As such, assuming linear coupling, the nematic order at the interface is approximately half the magnitude in the ordered phase. A typical value for inner lamellar structures found in the simulations of $q_{\text{lam}}\approx 2/3$ (implying α≈0.37), predicts the characteristic interface value $q_{c}=\alpha \phi_{\text{interface}}\approx 0.37$. Substituting these values into Eq. **7** yields $|W_{c}^{⊥}|\approx 2\kappa +0.2K$.

This prediction is validated by exploring phase space via numerical simulations. The transition to microphase separation is identified by the emergence of Bragg peaks in the structure factor at wavenumbers corresponding to a characteristic lamellar width $\lambda^{*}\approx 6$ (*SI Appendix*, Fig. S5). The critical anchoring is seen to be linear with κ, increasing with K and twice as large for parallel anchoring (Fig. 5). A linear regression of the simulated critical thresholds yields

[8a] $$\begin{array}{cc} |W_{c}^{⊥}{|}_{\text{sim}} & =2.001\kappa +0.138K+0.008, \end{array}$$

[8b] $$\begin{array}{cc} |W_{c}^{\|}{|}_{\text{sim}} & =3.987\kappa +0.253K+0.014, \end{array}$$

with a fit uncertainty of ±0.001 (enforcing zero intercepts leads to similar slopes but with a larger error, see *SI Appendix*). These results are in good agreement with the analytical estimate, particularly regarding the dominant κ contribution, where the simulations reproduce the predicted asymmetry and recover the factor of two difference between the regimes. Deviations in the K dependence may be attributed to simplifications in the model: the linear coupling approximation within the steep gradients of the interface, and our focus on a single lamellar domain, when the entire system exhibits a more complex texture where curvature of the lamellae (splay and bend of the director field) are present. Despite these differences, the overall trends support the proposed anchoring-driven mechanism of microphase separation.

### Elastic Stresses Template the Formation of Glassy Defect Patterns.

A notable feature of self-assembled lamellae is that it exhibits several undulations and long-lived defects in the lamellar ordering. A general mechanism to create undulations in layered systems—such as smectics or cholesterics—is the Helfrich-Herault instability, which creates deformations in response to geometric frustration, for instance arising from dilations or compression with respect to the thermodynamically favored spacing (33).

However, this is unlikely to be the responsible mechanism of undulations in the phase-separated stripes, as initializing the system with gaps between uniformly spaced layers leads to highly heterogeneous patterns in steady state, suggesting that there is no thermodynamic driving toward even layering (Movies S7 and S8). In other words, the lamellar phase has an unusually small compression modulus, so that it should not undergo a Helfrich-Hurault instability. Although the system does not favor evenly spaced layering, the layer width itself is self-selected and well-defined, as configurations initialized with different widths evolve toward a characteristic thickness (Movie S9).

Inspection of the kinetic pathways through which the undulating patterns form (Movies S7–S9) suggests that pattern selection is instead strongly dependent on initial random seeding due to the initial defect dynamics. The defects, which are formed early on, due to the noise in the initial configuration, result in elongated voids as isotropic droplets with opposite effective topological charge annihilate. Charged droplets which do not annihilate nucleate defects, such as smectic disclinations (Fig. 5; red *Inset*) or lamellar onions (Fig. 5; blue *Inset*). Following formation, such defects are kinetically arrested against coalescence, as coalescence would require large scale layer rearrangement, or tunneling of voids through layers. The spontaneous formation of onion patterns is remarkable, as in smectic phases these patterns normally require spontaneous curvature (22) or shear (34).

## Discussion and Conclusion

In summary, we have combined theory, simulations, and experiments to study the phase separation and pattern formation of a lyotropic liquid crystal composed of a mixture of a nematic and an isotropic fluid, under conditions for which there is no known underlying molecular attraction between the species promoting demixing. On one hand, this minimal model is expected to be broadly applicable to lyotropic nematic mixture. On the other hand, it provides a meaningful starting point to analyze our experimental system, the well-studied chromonic SSY-water mixture, which features a broad phase coexistence region in the phase diagram in the absence of a well-defined mechanism to promote attraction between self-assembled stacks of SSY molecules (4). While chromonics are notorious for their low twist elastic constant, twist does not appear to play a role in our quasi-2D geometry, hence our minimal single elastic constant approximation is reasonable in this context.

Our simulations and theory show that phase separation can emerge in lyotropics purely due to the coupling between local nematogen density and orientational order. This provides a robust physical mechanism to explain the large region of phase coexistence which is experimentally observed. Essentially, demixing arises because forming an isotropic droplet in a nematic domain is, in a large region of parameter space, more favorable thermodynamically than defect formation.

The kinetic pathway accompanying demixing and the growth of nematic and isotropic domains depends on the balance between elasticity and interfacial stresses, which is controlled by an elastocapillary number Ec. When elasticity dominates (small Ec), demixing is mainly driven by the coarsening of topological defects in the nematic phase, which is observed in both experiments and simulations. Defects nucleate voids, which become isotropic droplets inside nematic domains. As mentioned above, these voids are less costly thermodynamically than the elastic deformations near defect cores, however these droplets retain a net topological charge, which drives (through elasticity) coarsening and merging of droplets with opposite sign of the topological charge. Coalescence arrests once the remaining droplets have negligible topological charge. If, instead, the surface tension between the two components of the lyotropic mixture is sufficiently high (large Ec), coarsening proceeds indefinitely, resulting in macroscopic phase separation, in a similar manner to model B or model H dynamics, which describe phase separation in binary mixtures of isotropic fluids (35, 36).

A notable prediction of our minimal model, which is verified experimentally in SSY-water lyotropic mixtures, is the self-assembly of a supramolecular lamellar phase composed of nematically ordered microdomains. In this sense, this phase may be viewed as a form of *supramolecular smectic*, as it consists of elongated, microphase-separated nematic domains organized into layer-like arrangements, rather than molecular layers. Anchoring provides a general and robust mechanism to self-assembly microphase-separated stripes, because it acts as a surfactant thermodynamically, leading to an effectively negative surface tension which drives microphase separation. The stripe phase is observed experimentally near the transition to the isotropic mixed phase.

Both experiments and simulations show that the lyotropic lamellar phase is unusual, in that it contains multiple undulations and defects, such as lamellar onions. Undulations and onions normally appear due to shear (34, 37), geometric frustration (33), or spontaneous layer curvature (22). In our case, these defects are seeded by the topologically charged voids arising early on during the dynamics, which, once trapped in the gaps between the nematic layers, lose mobility leading to kinetically frozen or glassy defect patterns. This mechanism of formation substantially enhances the pattern formation potential of these lyotropic materials, as different initial conditions can in principle seed and template the formation of different glassy defect profiles. On this matter, the effect of dimensionality and anchoring at the cell plates will be the focus of future experimental and simulation work. This is interesting for future applications, especially as our experimental formulation of this material in terms of chromonic SSY is both widely available and biocompatible (38), raising the possibility of building multistable soft glasses via sustainable self-assembly.

## Materials and Methods

This section presents the hydrodynamic model used in this work. The dynamics of the lyotropic nematic mixture are described by three coarse-grained fields: i) the fluid velocity v(r,t), ii) the tensor order parameter Q(r,t) (39) describing the orientational order of the nematic liquid crystal, and iii) the scalar order parameter ϕ(r,t) representing the mixture composition. Furthermore, we detail the preparation of the SSY lyotropic phase, as well as the methods used to construct the phase diagram and perform the experiments.

### Nematic Fluid.

The nematic liquid crystal is described by a tensor order parameter Q that encodes the alignment of rod-like molecules in the liquid. In the uniaxial approximation, Q is a traceless symmetric tensor of the form $Q_{\alpha \beta }=q\left(n_{\alpha }n_{\beta }-\delta_{\alpha \beta }/3\right)$, where Greek indices denote Cartesian coordinates. Here, n is the director field representing the average molecular orientation, while the scalar q quantifies the degree of local order. The magnitude q is proportional to the largest eigenvalue of Q and is bounded by 0≤q≤3/2.

The physical properties of the liquid crystal are captured by a free energy F that depends on the order parameter Q. The most common form for its density is the Landau de-Gennes free energy density

[9] $$\begin{array}{c} \begin{array}{cc} f_{\mathrm{LC}}= & \frac{A_{0}}{2}\left(1-\frac{\gamma }{3}\right)Q_{\alpha \beta }^{2}-\frac{A_{0}\gamma }{3}Q_{\alpha \beta }Q_{\beta \gamma }Q_{\gamma \alpha }\\ & +\frac{A_{0}\gamma }{4}{\left(Q_{\alpha \beta }^{2}\right)}^{2}+\frac{K}{2}{\left(\partial_{\alpha }Q_{\beta \gamma }\right)}^{2}, \end{array} \end{array}$$

where Einstein summation convention is implied. The polynomial terms in Eq. **9** account for the bulk free energy density, where $A_{0}$ is a positive constant that sets the energy scale. The parameter γ acts as an effective inverse temperature as increasing γ (cooling) drives the isotropic–nematic transition, with the nematic phase stable for $\gamma >\gamma_{c}=2.7$. The final term accounts for the elastic energy associated with the director field. We adopt the one-constant approximation, setting the energy costs for splay, bend, and twist distortions as equivalent, and governed by the constant K.

The evolution of the Q tensor is given by the Beris-Edwards equation (40)

[10] $$\begin{array}{c} \left(\partial_{t}+v\cdot \nabla \right)Q-S=\Gamma H, \end{array}$$

where Γ is a collective rotational diffusion constant and H is the molecular field, given by

[11] $$\begin{array}{c} H=-\frac{\delta F}{\delta Q}+\frac{I}{3}\;\text{Tr}\frac{\delta F}{\delta Q}, \end{array}$$

where I is the identity matrix and Tr denotes the tensorial trace. The first term in Eq. **10** is the material derivative of Q describing its time evolution under advection by a fluid with velocity v. The second term in Eq. **10** describes how the molecules of the liquid fluid rotate and stretch in response to flow gradients (40), defined explicitly as

[12] $$\begin{array}{c} \begin{array}{cc} S= & \left(\xi D+\omega \right)\cdot \left(Q+\frac{I}{3}\right)+\left(Q+\frac{I}{3}\right)\cdot \left(\xi D-\omega \right)\\ & -2\xi \left(Q+\frac{I}{3}\right)\;\text{Tr}\left(Q\cdot \nabla v\right). \end{array} \end{array}$$

Here, $D=(\nabla v+\nabla v^{T})/2$ and $\omega =(\nabla v-\nabla v^{T})/2$ are the symmetric and antisymmetric parts of the velocity gradient tensor ∇v, representing the rate of strain and vorticity, respectively. The tumbling parameter ξ determines the molecular shape anisotropy, where positive values correspond to rod-like molecules and negative values to disk-like ones. The term on the right-hand side of Eq. **10** describes the relaxation of the tensorial order parameter toward the free energy minimum.

Last, the fluid velocity evolves according to the Navier–Stokes equation

[13] $$\begin{array}{c} \rho \left(\partial_{t}+u_{\beta }\partial_{\beta }\right)u_{\alpha }=-\partial_{\alpha }p_{0}+\partial_{\beta }\Pi_{\alpha \beta }+\eta \partial_{\beta }^{2}u_{\alpha }-\phi \partial_{\alpha }\mu , \end{array}$$

under the incompressibility condition $\partial_{\alpha }u_{\alpha }=0$, where μ is the chemical potential (see later). In Eq. **13** ρ is the fluid density, η is an isotropic viscosity and $p_{0}$ the hydrodynamic pressure. The stress that arises from the liquid crystal deformations $\Pi_{\alpha \beta }$ is given by

[14] $$\begin{array}{c} \begin{array}{cc} \Pi_{\alpha \beta }= & 2\xi \left(Q_{\alpha \beta }+\frac{1}{3}\delta_{\alpha \beta }\right)Q_{\gamma \epsilon }H_{\gamma \epsilon }-\xi \left(Q_{\gamma \beta }+\frac{1}{3}\delta_{\gamma \beta }\right)H_{\alpha \gamma }\\ & -\xi \left(Q_{\alpha \gamma }+\frac{1}{3}\delta_{\alpha \gamma }\right)H_{\gamma \beta }-\partial_{\alpha }Q_{\gamma \nu }\frac{\partial f}{\partial (\partial_{\beta }Q_{\gamma \nu })}\\ & +Q_{\alpha \gamma }H_{\gamma \beta }-H_{\alpha \gamma }Q_{\gamma \beta }. \end{array} \end{array}$$

### Binary Mixture.

The local composition of the binary mixture is given by the compositional order parameter ϕ, such that ϕ=0 corresponds to an isotropic fluid and ϕ=2 to a pure nematic liquid crystal. The free energy density that describes a non-phase-separating mixture is given by

[15] $$\begin{array}{c} f_{\phi }=\frac{a}{2}\phi^{2}+\frac{\kappa }{2}{\left(\partial_{\alpha }\phi \right)}^{2}+WQ_{\alpha \beta }\partial_{\alpha }\phi \partial_{\beta }\phi , \end{array}$$

where a is a positive constant. The second term in Eq. **15** penalizes spatial gradients of the order parameter, thereby defining the interfacial energy. The two terms a and κ scale the bulk and gradient contributions in the free energy, and contribute to the surface tension σ, and interface thickness between different emerging phases. The third term in Eq. **15**) couples the director field to compositional gradients, enforcing preferential anchoring at interfaces. The anchoring strength W determines whether the anchoring is homeotropic (W<0) or planar (W>0).

Similarly to what was done in a previous work (26), we define a linear dependence of γ (from Eq. **9**) on the local nematic composition

[16] $$\begin{array}{c} \gamma =\gamma_{0}+\Delta \;\phi , \end{array}$$

where $\gamma_{0}$ is a bare coupling parameter and Δ, the coupling parameter, a positive constant.

Finally, the evolution of the compositional order ϕ follows a Cahn–Hilliard equation

[17] $$\begin{array}{c} \partial_{t}\phi +v\cdot \nabla \phi =M\nabla^{2}\mu , \end{array}$$

where M is the mobility and μ=δF/δϕ is the chemical potential.

This work uses a 2D hybrid lattice Boltzmann (LB) scheme (41), solving Eq. **10** and Eq. **17** with finite differences, while Eq. **13** was integrated using the LB approach. The fixed parameters chosen to more closely match the experimental results are, unless previously stated otherwise, Δ=1, $A_{0}=0.25$, K=0.01, Γ=1, η=1.7, a=0.01, κ=0.01, and M=1. In our simulations, ξ=0.7, corresponding to a flow-aligning regime in the nematic phase.

### Topological Charge Density.

For our system, we compute the topological charge density $q_{t}$ as (42)

[18] $$\begin{array}{c} q_{t}\left(x,y\right)=\frac{1}{2\pi }\left(\partial_{x}Q_{\mathrm{xx}}\partial_{y}Q_{\mathrm{xy}}-\partial_{x}Q_{\mathrm{xy}}\partial_{y}Q_{\mathrm{xx}}\right). \end{array}$$

It is important to note that Eq. **18** is well defined for a lyotropic mixture as well as a single-fluid nematic, because the Q tensor is defined at each point, and both in nematic and isotropic regions. As a result, because in our mixture the system can create droplets and voids, the topological charge of a region can delocalize or spread over interfaces rather than being localized at singularities as normally occurs in a single-phase nematic.

We note for completeness that $q_{t}$ is the absolute value of the zz component of the recently introduced disclination tensor (43–45),

[19] $$\begin{array}{c} D_{\mathrm{ij}}=\epsilon_{i\mu \nu }\epsilon_{\mathrm{jlk}}\partial_{l}Q_{\mu \alpha }\partial_{k}Q_{\nu \alpha }, \end{array}$$

where i,j,k,α,μ,ν are tensor indices and where the Einstein summation convention of repeated indices has been used.

### Preparation of Sunset Yellow Lyotropic Phase.

Sunset Yellow FCF (SSY) purchased from Sigma Aldrich, with purity >90%, was further purified following the method of Horowitz et al. (8). This involved dissolving SSY in Milli-Q water and adding ethanol, causing it to precipitate. The precipitate was then isolated with filter paper, and dried in a vacuum oven. This procedure was carried out twice before being used in experiments. Samples of lyotropic SSY were created by adding dried, purified SSY to Milli-Q water at 29 wt%, and heated at around 50 °C until the SSY was dissolved completely.

### Temperature Ramp Experiments with Sunset Yellow.

Lyotropic SSY was loaded into a 20 μm-thick cell, which was then sealed prior to temperature ramp experiments. Temperature ramps were performed using an Instec mK1000, with the temperature increased at rates of 0.1, 0.2, and 0.5 °C min^−1^. For black-and-white time-lapse imaging, samples were observed using a Nikon ECLIPSE TE300 microscope, with movies recorded using a Mikrotron EoSens MC1362 high-speed camera. Color still images were acquired using an Olympus BH2-UMA microscope, with images captured using a QImaging MicroPublisher 3.3 RTV camera. All samples were viewed between crossed polarizers.

## Supplementary Material

Appendix 01 (PDF)

Movie S1. Phase separation and coarsening starting from a random, noisy initial configuration with no surface tension (*κ* = 0) or anchoring (*W* = 0). Zoomed out animation of the full 128×128 system presented in Fig.3A of the main text. The colour map represents the local compositional phase, *ϕ*, while the overlaid lines indicate the nematic director field, with line length proportional to the local degree of orientational order. Global compositional phase is fixed at *ϕ*_0_ = 1.0 and the bare coupling parameter at *γ*_0_ = 2.0. As time passes, defects annihilate, irregular droplets form and domains coarsen over time due to Ostwald ripening.

Movie S2. Phase separation and coarsening starting from a random, noisy initial configuration with surface tension (*κ* = 0.01) and no anchoring (*W* = 0). Zoomed out animation of the full 128×128 system presented in Fig.3B of the main text. The colour map represents the local compositional phase, *ϕ*, while the overlaid lines indicate the nematic director field, with line length proportional to the local degree of orientational order. Global compositional phase is fixed at *ϕ*_0_ = 1.0 and the bare coupling parameter at *γ*_0_ = 2.0. As time passes isotropic droplets form from the annihilation of topological defects. Droplets are rounder in shape and coalesce, in resemblance to the experiments.

Movie S3. Mixture of SSY at 28 wt% undergoing a temperature ramp from 26 to 43 °C. Snapshots from this movie are included in Fig.3 of the main text and Supp. Fig. 2. As the temperature increases, defects annihilate and isotropic droplets emerge and coarsen over time until eventually all of the mixtures becomes isotropic.

Movie S4. Mixture of SSY at 28 wt% undergoing a temperature ramp from 25 (nematic phase) to 45 °C (isotropic phase). In the coexistence regime the nematic phase forms lamella structures of well defined thickness. A snapshots from this movie is included in Fig.4A of the main text.

Movie S5. Time evolution of a simulation with *ϕ*_0_ = 1.0, *γ*_0_ = 2.0, *κ* = 0.01 and *W* = −0.03 (homeotropic anchoring) starting from a random and noisy configuration. Defect dynamics are shown: +1/2 (yellow) and −1/2 (blue) defects appear, move, and annihilate to relax the lamellar structure. However, full annihilation does not occur; anchoring stabilises microphase separation. This movie relates to panel D from Fig.4 of the main text, in this case, defects are shown reappearing transiently. System size: 50 × 50.

Movie S6. Time evolution of a simulation with *ϕ*_0_ = 1.0, *γ*_0_ = 2.0, *κ* = 0.01 and *W* = −0.03 (homeotropic anchoring) starting from a random and noisy configuration. Defect dynamics are shown: +1/2 (yellow) and −1/2 (blue) defects appear, move, and annihilate to relax the lamellar structure. However, full annihilation does not occur; anchoring stabilises microphase separation. This movie relates to panel E from Fig.4 of the main text, in this case, defects are shown to act as a source of the nematic layers. System size: 50 × 50.

Movie S7. Simulation initialised with a horizontally aligned lamellar pattern subject to additive noise. The initial bands are uniformly distributed throughout the domain, with widths comparable with those observed in Fig. 4B and Supplementary Movies 5 and 6 (λ* ≈ 6). The stability of the *lamella* width indicates that the system has reached an equilibrium state. Simulation parameters correspond to those of Fig. 4B: surface tension *κ* = 0.01, homeotropic anchoring *W* = −0.03, global composition *ϕ*_0_ = 1.0, and bare coupling constant *γ*_0_ = 1.0.

Movie S8. Horizontal lamellar pattern with heterogeneous spacing and additive noise. Band widths correspond to those of Fig. 4B (*λ* ≈ 6). The persistence of unoccupied regions highlights the unusually low layercompression modulus of the self-assembled smectics. Simulation parameters correspond to those of Fig. 4B: surface tension *κ* = 0.01, homeotropic anchoring *W* = −0.03, global composition *ϕ*_0_ = 1.0, and bare coupling constant *γ*_0_ = 1.0.

Movie S9. Simulation initialised with a diagonal lamellar pattern subject to additive noise. The initial structures evolve into thinner lamellae with widths comparable to those observed in Fig. 4B, D and E, and Supplementary Movies 5 and 6. Simulation parameters correspond to those of Fig. 4B: surface tension *κ* = 0.01, homeotropic anchoring *W* = −0.03, global composition *ϕ*_0_ = 1.0, and bare coupling constant *γ*_0_ = 1.0.

## Data Availability

Simulations data have been deposited in GitHub (https://github.com/GNegroLab/Spontaneous-phase-separation-and-pattern-formation-in-a-lyotropic-nematic-mixture) (46).

## References

[1] I. Dierking, A. Martins, F. Neto, Novel trends in lyotropic liquid crystals. Crystals **10**, 604 (2020).

[2] H. Wang , Liquid crystal biosensors: Principles, structure and applications. Biosensors **12**, 639 (2022). 36005035 10.3390/bios12080639 PMC9406233

[3] Y. Saadat, O. Q. Imran, C. O. Osuji, R. Foudazi, Lyotropic liquid crystals as templates for advanced materials. J. Mater. Chem. A **9**, 21607–21658 (2021).

[4] H. S. Park , Self-assembly of lyotropic chromonic liquid crystal sunset yellow and effects of ionic additives. J. Phys. Chem. B **112**, 16307–16319 (2008). 19368025 10.1021/jp804767z

[5] J. Lydon, Chromonic liquid crystalline phases. Liq. Cryst. **38**, 1663–1681 (2011).

[6] S. V. Shiyanovskii , Lyotropic chromonic liquid crystals for biological sensing applications. Mol. Cryst. Liq. Cryst. **434**, 259/[587]–270/[598] (2005).

[7] D. Edwards , Chromonic liquid crystal formation by edicol sunset yellow. J. Phys. Chem. B **112**, 14628–14636 (2008). 18729399 10.1021/jp802758m

[8] V. R. Horowitz, L. A. Janowitz, A. L. Modic, P. A. Heiney, P. J. Collings, Aggregation behavior and chromonic liquid crystal properties of an anionic monoazo dye. Phys. Rev. E **72**, 041710 (2005). 10.1103/PhysRevE.72.041710 16383405

[9] L. Joshi, S. W. Kang, D. M. Agra-Kooijman, S. Kumar, Concentration, temperature, and ph dependence of sunset-yellow aggregates in aqueous solutions: An x-ray investigation. Phys. Rev. E **80**, 041703 (2009). 10.1103/PhysRevE.80.041703 19905321

[10] Y. A. Nastishin , Optical characterization of the nematic lyotropic chromonic liquid crystals: Light absorption, birefringence, and scalar order parameter. Phys. Rev. E **72**, 041711 (2005). 10.1103/PhysRevE.72.041711 16383406

[11] A. Matsuyama, R. Evans, M. Cates, Non-uniformities in polymer/liquid crystal mixtures: I. Interfacial tension. Eur. Phys. J. E **9**, 79–87 (2002). 15010933 10.1140/epje/i2002-10061-9

[12] J. S. Lintuvuori, K. Stratford, M. Cates, D. Marenduzzo, Mixtures of blue phase liquid crystal with simple liquids: Elastic emulsions and cubic fluid cylinders. Phys. Rev. Lett. **121**, 037802 (2018). 30085823 10.1103/PhysRevLett.121.037802

[13] F. Guillén-González, M. Á. Rodríguez-Bellido, G. Tierra, Linear unconditional energy-stable splitting schemes for a phase-field model for nematic–isotropic flows with anchoring effects. *Int. J. Numer. Methods Eng*. **108**, 535–567 (2016).

[14] R. Meister, H. Dumoulin, M. A. Hallé, P. Pieranski, The anchoring of a cholesteric liquid crystal at the free surface. J. Phys. **6**, 827–844 (1996). 10.1103/physreve.54.3771 9965528

[15] H. Stark, Physics of colloidal dispersions in nematic liquid crystals. Phys. Rep. **351**, 387–474 (2001).

[16] I. Muševič, M. Škarabot, U. Tkalec, M. Ravnik, S. Žumer, Two-dimensional nematic colloidal crystals self-assembled by topological defects. Science **313**, 954–958 (2006). 16917058 10.1126/science.1129660

[17] I. I. Smalyukh, Liquid crystal colloids. Annu. Rev. Condens. Matter Phys. **9**, 207–226 (2018).

[18] O. D. Lavrentovich, Active colloids in liquid crystals. Curr. Opin. Colloid Interface Sci. **21**, 97–109 (2016).

[19] M. O’Keefe , Templated self-assembly of gold nanoparticles in smectic liquid crystals confined at 3d printed curved surfaces. Nanoscale **17**, 20351–20364 (2025). 40856036 10.1039/d4nr02539c

[20] G. Negro , Topology controls flow patterns in active double emulsions. Nat. Comm. **16**, 1412 (2025). 10.1038/s41467-025-56236-8 PMC11802772 39915471

[21] J. S. Lintuvuori , Colloidal templating at a cholesteric-oil interface: Assembly guided by an array of disclination lines. Phys. Rev. Lett. **110**, 187801 (2013). 23683244 10.1103/PhysRevLett.110.187801

[22] L. Ramos, D. Roux, P. D. Olmsted, M. Cates, Equilibrium onions? Eur. Lett. **66**, 888 (2004).

[23] T. Araki, H. Tanaka, Nematohydrodynamic effects on the phase separation of a symmetric mixture of an isotropic liquid and a liquid crystal. Phys. Rev. Lett. **93**, 015702 (2004).

[24] N. Sulaiman, D. Marenduzzo, J. Yeomans, Lattice Boltzmann algorithm to simulate isotropic-nematic emulsions. Phys. Rev. E **74**, 041708 (2006). 10.1103/PhysRevE.74.041708 17155079

[25] M. M. T. da Gama, R. C. Coelho, Phase separation in mixtures of nematic and isotropic fluids. Annu. Rev. Condens. Matter Phys. **17**, 115–135 (2025).

[26] R. Assante, D. Corbett, D. Marenduzzo, A. Morozov, Active turbulence and spontaneous phase separation in inhomogeneous extensile active gels. Soft Matter **19**, 189–198 (2023). 36503973 10.1039/d2sm01188c

[27] I. Chuang, R. Durrer, N. Turok, B. Yurke, Cosmology in the laboratory: Defect dynamics in liquid crystals. Science **251**, 1336–1342 (1991). 17816188 10.1126/science.251.4999.1336

[28] A. J. Bray, Theory of phase-ordering kinetics. Adv. Phys. **43**, 357–459 (1994).

[29] A. Morozov, J. Fraaije, Phase behavior of block copolymer melts with arbitrary architecture. J. Chem. Phys. **114**, 2452–2465 (2001).

[30] S. Brazovskii, Phase transition of an isotropic system to a nonuniform state. Sov. Phys. JETP **41**, 85 (1975).

[31] G. Gonnella, E. Orlandini, J. Yeomans, Spinodal decomposition to a lamellar phase: Effects of hydrodynamic flow. Phys. Rev. Lett. **78**, 1695 (1997).

[32] A. Xu, G. Gonnella, A. Lamura, Morphologies and flow patterns in quenching of lamellar systems with shear. Phys. Rev. E **74**, 011505 (2006). 10.1103/PhysRevE.74.011505 16907098

[33] C. Blanc , Helfrich-hurault elastic instabilities driven by geometrical frustration. Rev. Mod. Phys. **95**, 015004 (2023).

[34] E. Van der Linden, W. Hogervorst, H. Lekkerkerker, Relation between the size of lamellar droplets in onion phases and their effective surface tension. Langmuir **12**, 3127–3130 (1996).

[35] P. M. Chaikin, T. C. Lubensky, T. A. Witten, Principles of Condensed Matter Physics (Cambridge University Press, Cambridge, 1995), vol. 10.

[36] M. E. Cates, E. Tjhung, Theories of binary fluid mixtures: From phase-separation kinetics to active emulsions. J. Fluid Mech. **836**, P1 (2018).

[37] J. Arrault, W. Poon, M. Cates, Structure and rheology of composite soft solids: Particles in lamellar phases. Phys. Rev. E **59**, 3242 (1999).

[38] V. P. Chavda , Lyotropic liquid crystalline phases: Drug delivery and biomedical applications. Int. J. Pharm. **647**, 123546 (2023). 37884213 10.1016/j.ijpharm.2023.123546

[39] P. G. de Gennes, J. Prost, *The Physics of Liquid Crystals* (Oxford University Press, Oxford, UK, ed. 2, 1993).

[40] A. N. Beris, B. J. Edwards, Thermodynamics of Flowing Systems with Internal Microstructure (Oxford University Press, 1994).

[41] L. N. Carenza, G. Gonnella, A. Lamura, G. Negro, A. Tiribocchi, Lattice Boltzmann methods and active fluids. Eur. Phys. J. E **42**, 81 (2019). 31250142 10.1140/epje/i2019-11843-6

[42] M. L. Blow, S. P. Thampi, J. M. Yeomans, Biphasic, lyotropic, active nematics. Phys. Rev. Lett. **113**, 248303 (2014). 25541809 10.1103/PhysRevLett.113.248303

[43] L. C. Head , Majorana quasiparticles and topological phases in 3d active nematics. Proc. Natl. Acad. Sci. U.S.A. **121**, e2405304121 (2024). 39700144 10.1073/pnas.2405304121 PMC11670186

[44] N. Johnson , Clifford algebras and liquid crystalline fermions. Phys. Rev. X **15**, 041052 (2025).

[45] C. D. Schimming, J. Viñals, Singularity identification for the characterization of topology, geometry, and motion of nematic disclination lines. Soft Matter **18**, 2234–2244 (2022). 35234228 10.1039/d1sm01584b

[46] A. Bensabat, G. Negro, Spontaneous-phase-separation-and-pattern-formation-in-a-lyotropic-nematic-mixture. GitHub. https://github.com/GNegroLab/Spontaneous-phase-separation-and-pattern-formation-in-a-lyotropic-nematic-mixture. Deposited 18 June 2026. 10.1073/pnas.2604649123 PMC13367829 42424425

