# Supplementary material for: Spontaneous phase separation and pattern formation in a lyotropic nematic mixture
Source: Proc Natl Acad Sci U S A. 2026 Jul 9;123(28):e2604649123. doi: 10.1073/pnas.2604649123 (PMC13367829; doi:10.1073/pnas.2604649123)
Supplement: Supplementary file 1 — Appendix 01 (PDF) [file pnas.2604649123.sapp.pdf]

Supplementary Information

Spontaneous phase separation and pattern formation in a lyotropic nematic mixture

A. Bensabat, Ó. Skelton, J. Arlt, M. Bjelogrlic, D. Marenduzzo, G. Negro, T. N. Shendruk, T. A. Wood

June 15, 2026

1 Identification of a phase separated regime

To build the phase diagram from the simulations and identify the phase separated regimes, the average nematic order and the Binder cumulants were computed.

The local nematic order corresponds to the highest eigenvalue of the **Q**-tensor in each point in space. The average nematic order $\langle q \rangle$, is the average of the local one over all the points. In Fig.2B of the main text, $\langle q \rangle$ is plotted for different values of γ_0 and ϕ_0. There is a clear transition from the isotropic phase ($\langle q \rangle \approx 0$) to the coexistence region ($0.2 < \langle q \rangle < 0.5$) and nematic ($\langle q \rangle \approx 2/3$).

The Binder cumulant is computed from

$$U_\phi = 1 - \frac{\langle \phi \rangle^4}{3\langle \phi^2 \rangle^2}, \tag{1}$$

where the averages are taken over all the points in space for a given configuration. According to (1), a Binder cumulant under 2/3 is indicative of a coexistence of different phases. In Fig.2C of the main text values of the Binder cumulant of $0.2 < U_\phi < 0.5$ confirm the coexistence of nematic and isotropic phases.

2 Calculations for the common tangent construction

Let us consider the free energy of the binary mixture given by Eq.(7) and Eq.(13) of the main text. The full free energy density is

$$\begin{aligned} f = \frac{A_0}{2}\left(1 - \frac{\gamma(\phi)}{3}\right) Q_{\alpha\beta}^2 - \frac{A_0\gamma(\phi)}{3} Q_{\alpha\beta}Q_{\beta\gamma}Q_{\gamma\alpha} \frac{A_0\gamma(\phi)}{4}(Q_{\alpha\beta}^2)^2 + \frac{K}{2}(\partial_\alpha Q_{\beta\gamma})^2 \\ + \frac{a}{2}\phi^2 + \frac{\kappa}{2}(\partial_\alpha \phi)^2 + W Q_{\alpha\beta}\partial_\alpha\phi\partial_\beta\phi. \end{aligned} \tag{2}$$

To determine the phase boundaries of the system, we consider a homogeneous configuration of unvarying composition and uniformly aligned nematic phase. In this state, the third (elastic energy), fifth (interfacial energy), and seventh (anchoring) terms vanish, and the homogeneous free energy density depends only on the nematic order q and the composition ϕ.

Given that the nematic order parameter is given by $Q_{\alpha\beta} = q(n_\alpha n_\beta - \delta_{\alpha\beta}/3)$, the homogeneous free energy simplifies to:

$$f_{\mathrm{hom}}(q,\phi) = \frac{A_0}{3}\left(1 - \frac{\gamma(\phi)}{3}\right) q^2 - \frac{2A_0\gamma(\phi)}{27} q^3 + \frac{A_0\gamma(\phi)}{9} q^4 + \frac{a}{2}\phi^2, \tag{3}$$

where the total free energy is minimised for the nematic order parameter

$$q_{\min} = \begin{cases} 0 & \text{if } \gamma \le \gamma_c, \\ \frac{1}{4}\left(1 + \sqrt{9 - 24/\gamma(\phi)}\right) & \text{if } \gamma \ge \gamma_c, \end{cases} \tag{4}$$

where $\gamma(\phi) = \gamma_0 + \Delta\phi$ and $\gamma_c = 2.7$. This free energy has two concavities (inset of Fig. 2D of the main text), thus defining a region where the second derivative of the free energy is negative, and the mixture is unstable.

The first derivative of the free energy is given by $\partial f_{\text{hom}}/\partial\phi = a\phi$ below γ_c and above γ_c by

$$\frac{\partial f_{\text{hom}}}{\partial \phi} = -\frac{A_0\Delta}{9}q^2 - \frac{2A_0\Delta}{27}q^3 + \frac{A_0\Delta}{9}q^4 + a\phi + \left[\frac{2A_0}{3}\left(1-\frac{\gamma(\phi)}{3}\right)q - \frac{2A_0\gamma(\phi)}{9}q^2 + \frac{4A_0\gamma(\phi)}{9}q^3\right]q'.$$

The second derivative is $\partial^2 f_{\text{hom}}/\partial\phi^2 = a$ below γ_c and above γ_c is

$$\frac{\partial^2 f_{\text{hom}}}{\partial \phi^2} = a + 2\left[-\frac{2A_0\Delta}{9}q - \frac{2A_0\Delta}{9}q^2 + \frac{4A_0\Delta}{9}q^3\right]q' + \left[\frac{2A_0}{3}\left(1-\frac{\gamma(\phi)}{3}\right) - \frac{4A_0\gamma(\phi)}{9}q + \frac{12A_0\gamma(\phi)}{9}q^2\right](q')^2 + \left[\frac{2A_0}{3}\left(1-\frac{\gamma(\phi)}{3}\right)q - \frac{2A_0\gamma(\phi)}{9}q^2 + \frac{4A_0\gamma(\phi)}{9}q^3\right]q'',$$

where

$$\frac{\partial q}{\partial \phi} = \frac{3\Delta}{\gamma(\phi)^2}\left(9-\frac{24}{\gamma(\phi)}\right)^{-1/2}, \qquad \frac{\partial^2 q}{\partial \phi^2} = \frac{-54\Delta^2(\gamma(\phi)-2)}{(\gamma(\phi))^4\left(9-\frac{24}{\gamma(\phi)}\right)^{3/2}}. \tag{5}$$

The phase diagram is built by varying the total composition of the mixture ϕ, and the bare coupling parameter γ_c and computing the respective spinodal and binodal regions numerically.

The spinodal region is defined by $\partial^2 f_{\text{hom}}/\partial\phi^2 < 0$ and is delimited by the points where the $\partial^2 f_{\text{hom}}/\partial\phi^2$ is discontinuous, $\phi_c = (2.7-\gamma_0)/\Delta$, and where $\partial^2 f_{\text{hom}}/\partial\phi^2 = 0$. The later point is estimated greedily by scanning through all the values of $\partial^2 f_{\text{hom}}/\partial\phi^2$ above the discontinuity and the selecting the value of ϕ that yields the $\partial^2 f_{\text{hom}}/\partial\phi^2$ closer to zero.

The binodal region is found by a greedy iterative method. Firstly a fine grid of ϕ is defined, and those grid points are split into two intervals, one for $\gamma < \gamma_c$ and one for $\gamma > \gamma_c$. Then, all pairs (ϕ_1, ϕ_2) formed by points of both intervals are sampled and the pair that satisfies the following condition

$$\min_{\{\phi_1,\phi_2\}}\left\{\left\|\left.\frac{\partial f_{\text{hom}}}{\partial\phi}\right|_{\phi_1} - \left.\frac{\partial f_{\text{hom}}}{\partial\phi}\right|_{\phi_2}\right\| + \left\|g(\phi_1)-g(\phi_2)\right\|\right\}, \tag{6}$$

is found. In Eq. (6), $g(\phi) = f_{\text{hom}}(\phi) - f'_{\text{hom}}(\phi)\phi$ is the y-intercept of the line tangent to the point $(\phi, f_{\text{hom}}(\phi))$. The pair that satisfies Eq. (6) defines the limits of the binodal region. The tangent line connecting the points $(\phi_1, f_{\text{hom}}(\phi_1))$ $(\phi_2, f_{\text{hom}}(\phi_2))$ represents the average free energy density of the phase-separated mixture, which is lower than that of the homogeneous state for any given average composition $\langle\phi\rangle$ lying between the binodals.

3 Effect of nematic elasticity

To assert whether the presented findings are general and present across different chromatic liquid crystal system, we have extended our numerical analysis by varying the nematic elastic constant K, while keeping fixed the values of ϕ_0 and γ_0 corresponding to the phase-separated regime discussed in the manuscript. These results are reported in Figs. 4 and 5.

These simulations show that the observed phenomenology is robust over a finite range of parameters, but that the morphology depends systematically on the competition between compositional demixing, interfacial effects, and nematic elasticity. In particular, for $\kappa = 0$, increasing K progressively suppresses fine-scale structure, leading to smoother and coarser domains, and eventually to a mixed state for sufficiently large K. When an explicit interfacial contribution is also included, the overall scenario remains qualitatively similar. When anchoring is also included, and for the parameter values at which stripe formation is observed, varying the elastic constant induces a morphological transition: the patterns evolve from more rounded and strongly deformed stripes to longer, more aligned stripe-like structures.

These trends are consistent with the interpretation that nematic elasticity effectively penalizes strongly distorted interfaces and therefore plays a role analogous to an additional surface tension. From this perspective, our results suggest that the behavior described in the manuscript is not tied to a single fine-tuned parameter choice, but instead reflects a more general mechanism arising from the coupling between composition and liquid-crystalline order. At the same time, the simulations also indicate that the location and extent of the different morphological regimes depend quantitatively on the elastic constants, so that the precise phase boundaries are expected to shift across different chromonic and, more broadly, lyotropic liquid-crystalline systems.

4 Critical anchoring

Let us again consider the free energy density of Eq. (2). To determine the anchoring strength for which phase separation is arrested, we examine a single lamellar nematic domain with a well-defined interface. We consider a region where the director field is spatially uniform ($\mathbf{n}(\mathbf{r}) = \mathbf{n}$) and coupled to the domain interface, such that $\mathbf{n} \cdot \nabla\phi/\|\nabla\phi\| = 0$ for planar anchoring and $\mathbf{n} \cdot \nabla\phi/\|\nabla\phi\| = 1$ for homeotropic anchoring. Under these conditions, the free energy simplifies to

$$f_{\text{lam}} = \frac{A_0}{3}\left(1 - \frac{\gamma(\phi)}{3}\right)q^2 - \frac{2A_0\gamma(\phi)}{27}q^3 + \frac{A_0\gamma(\phi)}{9}q^4 + \frac{K}{3}|\nabla q|^2 + \frac{a}{2}\phi^2 + \frac{\kappa}{2}|\nabla\phi|^2 + Wq\left[|\mathbf{n}\cdot\nabla\phi|^2 - \frac{1}{3}|\nabla\phi|^2\right]. \quad (7)$$

Assuming that the local nematic order is linearly coupled to the local concentration via $q(\mathbf{r}) \approx \alpha\phi(\mathbf{r})$, the free energy density can be recast to isolate the gradient contributions

$$f_{\text{lam}} \approx f_{\text{bulk}}(\phi, q) + \kappa_{\text{eff}}(\phi, q)(\nabla\phi)^2, \quad (8)$$

where an effective surface tension coefficient

$$\kappa_{\text{eff}} = \frac{\kappa}{2} + \frac{K\alpha^2}{3} + Wq\left(\frac{|\mathbf{n}\cdot\nabla\phi|^2}{|\nabla\phi|^2} - \frac{1}{3}\right) \quad (9)$$

appears. As described in the main text, macrophase separation is driven by a positive surface tension ($\kappa_{\text{eff}} > 0$), which drives the system to minimise interfacial area. However, the last term in κ_{eff} (Eq.(9)) is negative and so a transition occurs when this anchoring contribution renders κ_{eff} negative. The critical threshold for this transition ($\kappa_{\text{eff}} = 0$) is defined by

$$\left|W_c^{\perp}\right| = \frac{3}{2q}\left(\frac{\kappa}{2} + \frac{K\alpha^2}{3}\right), \quad W_c^{\parallel} = 2\left|W_c^{\perp}\right|, \quad (10)$$

for homeotropic and planar anchoring, respectively.

5 Lamella length scale characterisation

The lamellar patterns that emerge for high values of anchoring strength were characterised by measuring the oriented correlation function of the composition ϕ, and the structure factor.

The oriented correlation function is given by

$$G(\Delta r) = \frac{\langle\phi(\mathbf{r} + \hat{\mathbf{e}}\Delta r)\phi(\mathbf{r})\rangle}{\langle\phi\rangle}, \quad (11)$$

where the averages are spatial averages, taken over all the points in a given configuration, and $\hat{\mathbf{e}}$ is a unit versor. For the characterisation of the lammellar patterns shown in Fig. 3, the correlation function was sampled along the y direction (denoted as $G(y)$) and along the direction perpendicular to the *lamellae* (denoted as $G(r_\perp)$).

In panels A and B of Fig.3, $G(y)$ is shown for three specific configurations with homeotropic (pink lines), planar (blue lines) and no anchoring (bronze lines). Given that for these configurations the emergent lamellar patterns tendentially align themselves perpendicular to the y axis, the correlation function was sampled along y. In the absence of anchoring, $G(y)$ decays rapidly. In contrast, the oriented correlation functions for strong homeotropic and planar anchoring show a distinct decaying oscillatory trend. The high frequency oscillations are marks of the lamellar structure, with a wavelength of approximately $5 < \lambda^* < 8$ lattice units. In the case of homeotropic anchoring, there is not only a single decay in the correlation, but also an envelope whose wavelength ($\lambda \approx 50$) is related to the characteristic size of the domains of *lamella* that align in a single direction. The detection of an enveloping frequency for homeotropic anchoring is a mark of the more well defined structures that are found in this case in comparison to the planar anchroing case.

A further attempt at quantifying the length scales of the system was done by sampling the correlation function along the direction perpendicular to the *lamellae*. These values are plotted for the systems present in the inset of Fig. 3B in the presence of homeotropic (pink) and planar (blue) anchoring in Fig. 3F-H. The correlations agree with those of Fig. 3B, showing rapidly decaying oscillations for planar anchoring and enveloped ones in the homeotropic case. Panels G and H show semi-log plots of $|1 - G(r_\perp)|$ for homeotropic and planar anchoring respectively. From the distance between the peaks in Fig. 3G one can take $\lambda^* \approx 6$. However, as seen in the insets of panels A and B, though the *lamella* thickness is well defined, the spacing between *lamellae* is not. This coupled with the fact that

the *lamella* orientation is not spatially uniform makes it difficult to quantify precisely the length scales that are present in the system.

These length scales were further quantified via the structure factor (see Fig. 3C-E), computed as

$$S(\mathbf{k}) = \left| \int \mathrm{d}^2\mathbf{r} e^{-i\mathbf{k}\cdot\mathbf{r}} (\phi(\mathbf{r}) - \langle \phi(\mathbf{r}) \rangle) \right|^2. \tag{12}$$

Peaks emerge for frequencies of approximately $0.5 < k < 1.2$, which correspond to wavelengths of $5.2 < \lambda < 13$. These peaks are clearer for homeotropic anchoring (Fig. 3C) in which the lamellar structure is better defined. In contrast, for planar anchoring the peaks are more disperse and there is a bounding frequency for $k \approx 1.2$ that relates to the finer lengths scales in the system. Since the *lamella* are less pronounced in this case, we can also identify peaks at lower frequencies $k \approx 0.15 \implies \lambda \approx 42$ that relate to the domain sizes in which the *lamella* are pointing in the same direction. In the absence of anchoring, the structure factor peaks to the average size of isotropic droplets.

5.1 Detecting critical anchoring

To detect critical anchoring, the phase space of the surface tension coefficient κ and the nematic elastic constant K was explored for $\kappa \in \{0.001, 0.002, \cdots, 0.014\}$, $K \in \{0.005, 0.006, \cdots, 0.034\}$, $|W^{\perp}| \in \{0.001, 0.001, \cdots, 0.045\}$, and $|W^{\parallel}| \in \{0.001, 0.001, \cdots, 0.085\}$. For each pair (κ, K), the anchoring was gradually increased until the patterns were detected.

To detect the onset of pattern formation the structure factor was considered. As seen above (Fig. 3E), when planar anchoring is considered, the structure factor also exhibits pronounced peaks at lower frequencies, which are reminiscent of larger scale structures. Therefore, to detect the presence of the finer laminar structures, a mask was first applied to remove the lower frequency peaks (corresponding to $\lambda > 8$). Then, the maximum peak of the structure factor was computed, and for $|S(\mathbf{k})| > 10^4$ we considered patterns to have formed. The first value of W satisfying this condition was called the critical anchoring strength W_c.

By performing a bilinear fit of the obtained values of $W_c^{\perp}$ and $W_c^{\parallel}$ as functions of κ and K, the following fits are obtained:

$$|W_c^{\perp}| = 2.001\kappa + 0.138K + 0.008 \quad (\pm 0.001), \tag{13}$$

$$W_c^{\parallel} = 3.987\kappa + 0.253K + 0.014 \quad (\pm 0.001), \tag{14}$$

and by forcing a null $y-$intercept:

$$|W_c^{\perp}| = 2.401\kappa + 0.364K \quad (\pm 0.003), \tag{15}$$

$$W_c^{\parallel} = 4.681\kappa + 0.644K \quad (\pm 0.005). \tag{16}$$

6 Supplementary Figures

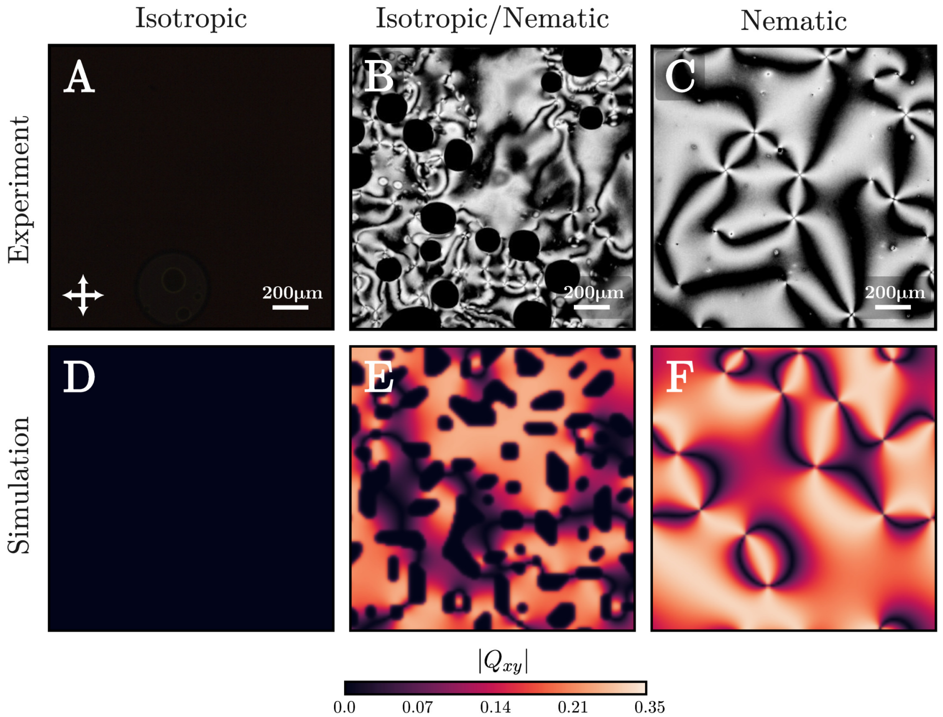

Figure 1: (A–C) Experimental images showing the isotropic (A), coexistence (B), and nematic (C) phases as the temperature decreases (γ_0 increases) and the nematic fluid concentration ϕ_0 increases. (D–F) Corresponding simulation snapshots illustrating the same sequence of phase behaviour and Schlieren patterns for a 128×128 system. The colour map represents the modulus of the $\mathbf{Q}$-tensor component $|Q_{xy}|$. Scale bars: 200 µm.

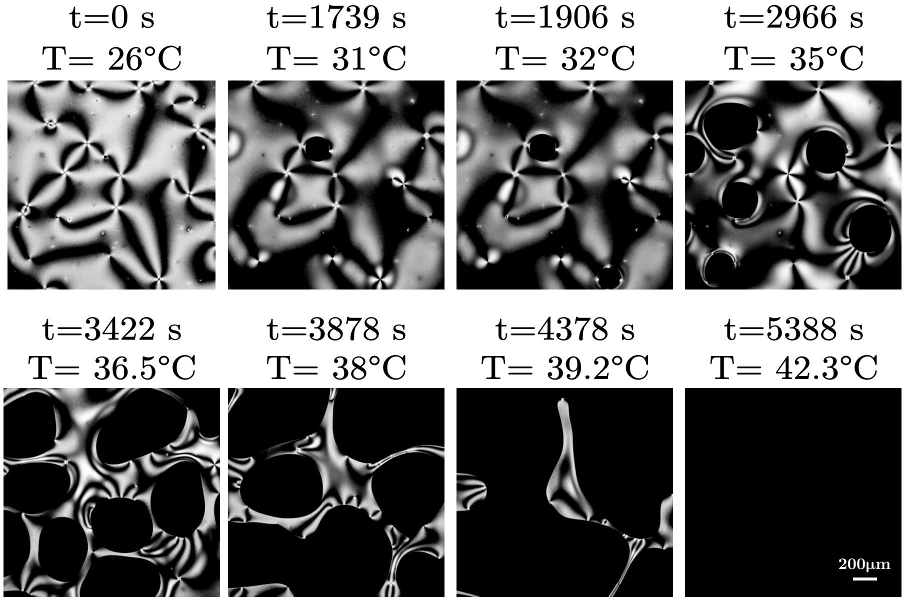

Figure 2: Transition from a fully nematic to fully isotropic phase of a mixture of SSY at 28 wt% undergoing a temperature ramp from 26.0 to 42.3 °C. Snapshots taken from Movie 1.

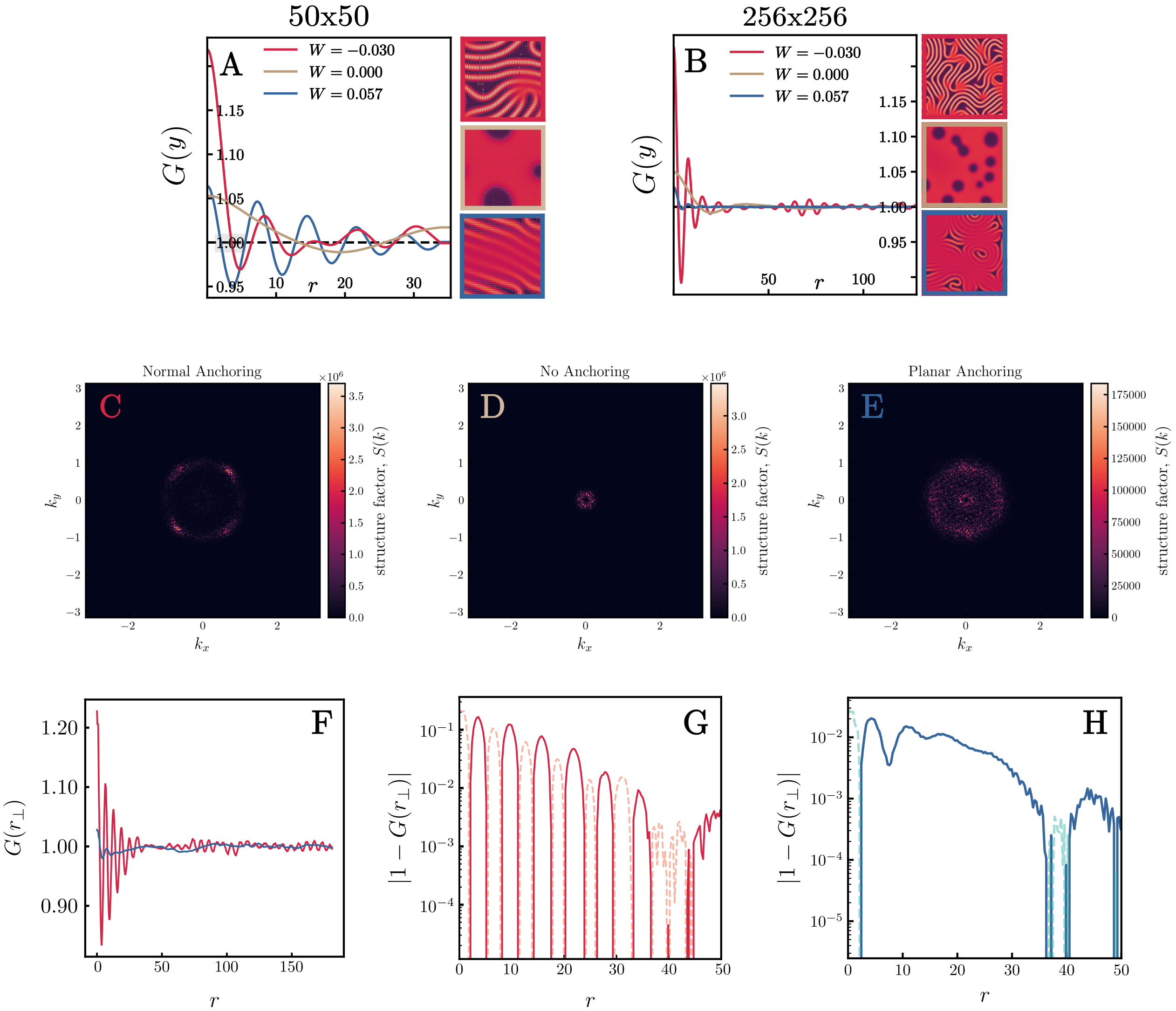

Figure 3: Structural analysis of the lamellar patterns with planar, homeotropic and zero anchoring. For all simulations $\phi_0 = 1.0$, $\gamma_0 = 2.0$ and $\kappa = 0.01$. A and B show the spatial correlation function for the nematic composition ϕ sampled in the y direction, $G(y)$, for homeotropic ($W = -0.030$), planar ($W = 0.057$) and zero anchoring $W = 0$, for two different system sizes. Side panels show the corresponding configurations. Panels C-E show the corresponding structure factor for the largest system size. Panel F shows the spatial correlation function sampled in direction parallel to the lamellar normals $G(r_\perp)$, for homeotropic (pink) and planar (blue) anchoring. G and H show the respective curves for $|1 - G(r_\perp)|$ (semi-log).

7 Movies

- Movie 1 - Phase separation and coarsening starting from a random, noisy initial configuration with no surface tension ($\kappa = 0$) or anchoring ($W = 0$). Zoomed out animation of the full 128×128 system presented in Fig.3A of the main text. The colour map represents the local compositional phase, ϕ, while the overlaid lines indicate the nematic director field, with line length proportional to the local degree of orientational order. Global compositional phase is fixed at $\phi_0 = 1.0$ and the bare coupling parameter at $\gamma_0 = 2.0$. As time passes, defects annihilate, irregular droplets form and domains coarsen over time due to Ostwald ripening.

- Movie 2 - Phase separation and coarsening starting from a random, noisy initial configuration with surface tension ($\kappa = 0.01$) and no anchoring ($W = 0$). Zoomed out animation of the full 128×128 system presented in Fig.3B of the main text. The colour map represents the local compositional phase, ϕ, while the overlaid

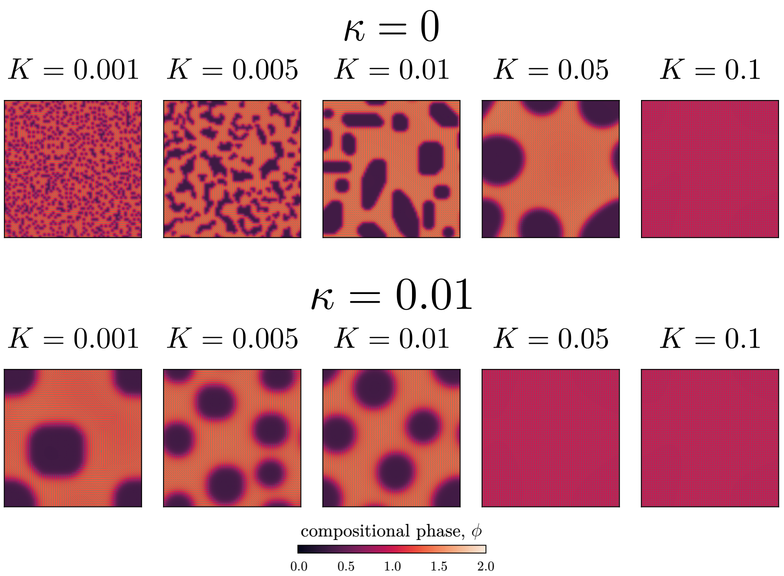

Figure 4: Snapshots of the system in the coexistence region, with $\gamma_0 = 2.0$ and $\phi_0 = 1.0$ for different values of nematic elasticity, K, and interfacial tension, κ.

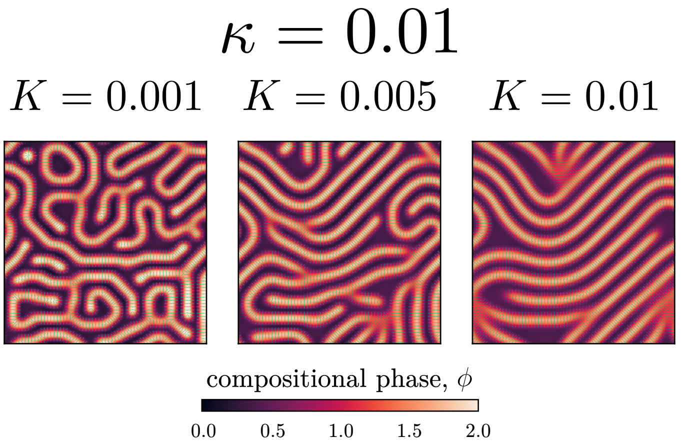

Figure 5: Snapshots of the system in the lamellar phase, with $\gamma_0 = 2.0$, $\phi_0 = 1.0$ and $W = -0.03$ (homeotropic anchoring) for different values of nematic elasticity, K.

lines indicate the nematic director field, with line length proportional to the local degree of orientational order. Global compositional phase is fixed at $\phi_0 = 1.0$ and the bare coupling parameter at $\gamma_0 = 2.0$. As time passes isotropic droplets form from the annihilation of topological defects. Droplets are rounder in shape and coalesce, in resemblance to the experiments.

- Movie 3 - Mixture of SSY at 28 wt% undergoing a temperature ramp from 26 to 43 °C. Snapshots from this movie are included in Fig.3 of the main text and Supp. Fig. 2. As the temperature increases, defects annihilate and isotropic droplets emerge and coarsen over time until eventually all of the mixtures becomes isotropic.

- Movie 4 - Mixture of SSY at 28 wt% undergoing a temperature ramp from 25 (nematic phase) to 45 °C (isotropic phase). In the coexistence regime the nematic phase forms *lamella* structures of well defined thickness. A snapshots from this movie is included in Fig.4A of the main text.

- Movie 5 - Time evolution of a simulation with $\phi_0 = 1.0$, $\gamma_0 = 2.0$, $\kappa = 0.01$ and $W = -0.03$ (homeotropic anchoring) starting from a random and noisy configuration. Defect dynamics are shown: $+1/2$ (yellow) and $-1/2$ (blue) defects appear, move, and annihilate to relax the lamellar structure. However, full annihilation does not occur; anchoring stabilises microphase separation. This movie relates to panel D from Fig.4 of the main text, in this case, defects are shown reappearing transiently. System size: 50×50.

- Movie 6 - Time evolution of a simulation with $\phi_0 = 1.0$, $\gamma_0 = 2.0$, $\kappa = 0.01$ and $W = -0.03$ (homeotropic anchoring) starting from a random and noisy configuration. Defect dynamics are shown: $+1/2$ (yellow) and $-1/2$ (blue) defects appear, move, and annihilate to relax the lamellar structure. However, full annihilation does not occur; anchoring stabilises microphase separation. This movie relates to panel E from Fig.4 of the main text, in this case, defects are shown to act as a source of the nematic layers. System size: 50×50.

- Movie 7 - Simulation initialised with a horizontally aligned lamellar pattern subject to additive noise. The initial bands are uniformly distributed throughout the domain, with widths comparable with those observed in Fig. 4B and Supplementary Movies 5 and 6 ($\lambda^* \approx 6$). The stability of the *lamella* width indicates that the system has reached an equilibrium state. Simulation parameters correspond to those of Fig. 4B: surface tension $\kappa = 0.01$, homeotropic anchoring $W = -0.03$, global composition $\phi_0 = 1.0$, and bare coupling constant $\gamma_0 = 1.0$.

- Movie 8 - Horizontal lamellar pattern with heterogeneous spacing and additive noise. Band widths correspond to those of Fig. 4B ($\lambda^* \approx 6$). The persistence of unoccupied regions highlights the unusually low layer-compression modulus of the self-assembled smectics. Simulation parameters correspond to those of Fig. 4B: surface tension $\kappa = 0.01$, homeotropic anchoring $W = -0.03$, global composition $\phi_0 = 1.0$, and bare coupling constant $\gamma_0 = 1.0$.

- Movie 9 - Simulation initialised with a diagonal lamellar pattern subject to additive noise. The initial structures evolve into thinner lamellae with widths comparable to those observed in Fig. 4B, D and E, and Supplementary Movies 5 and 6. Simulation parameters correspond to those of Fig. 4B: surface tension $\kappa = 0.01$, homeotropic anchoring $W = -0.03$, global composition $\phi_0 = 1.0$, and bare coupling constant $\gamma_0 = 1.0$.

All the movies are available here.
